# Are antenatal interventions effective in improving multiple health behaviours among pregnant women? A systematic review protocol

**DOI:** 10.1186/s13643-020-01453-z

**Published:** 2020-09-02

**Authors:** Jenna L. Hollis, Emma Doherty, Julia Dray, Danika Tremain, Mandy Hunter, Karen Takats, Christopher M. Williams, Henry Murray, Craig E. Pennell, Belinda Tully, John Wiggers, Justine B. Daly, Melanie Kingsland

**Affiliations:** 1 Hunter New England Population Health, Hunter New England Local Health District, Locked Bag 10, Wallsend, New South Wales 2287 Australia; 2 School of Medicine and Public Health, The University of Newcastle, Callaghan, New South Wales Australia; 3 Priority Research Centre for Health Behaviour, The University of Newcastle, Callaghan, New South Wales Australia; 4 Hunter Medical Research Institute, New Lambton Heights, New South Wales Australia; 5 Maternity and Gynaecology John Hunter Hospital, New Lambton Heights, New South Wales Australia

**Keywords:** SNAP, Smoking, Nutrition, Diet, Alcohol, Physical activity, Behaviour change, Prenatal, Pregnancy, Maternity

## Abstract

**Background:**

Maternal behaviours in pregnancy associated with adverse pregnancy, birth and health outcomes include tobacco smoking, poor nutrition, alcohol consumption and low physical activity, collectively referred to as the SNAP risk factors. Due to the high prevalence, co-occurrence and possible interactive health effects of such health behaviours in pregnancy, antenatal interventions that support pregnant women to improve multiple SNAP behaviours have a greater potential impact on the health outcomes of women and their children than interventions addressing single behaviours. The objective of this review is to determine the effectiveness of interventions delivered as part of antenatal care that aim to improve multiple SNAP behaviours among pregnant women.

**Methods:**

Seven electronic databases will be searched for potentially eligible studies. Eligible studies will include those where pregnant women are attending antenatal care. Studies that examine the effect of an intervention that addresses multiple SNAP behaviours (≥ 2 behaviours) during pregnancy and are delivered or instigated through antenatal care in a healthcare service will be included. Systematic reviews of randomised controlled trials (RCTs), RCTs, cluster RCTs, stepped-wedge RCTs and non-randomised control trials will be eligible. Studies that include a no-intervention control, wait-list control group, standard/usual care, or another active single behavioural intervention (e.g. addressing one behaviour only) will be considered. Two independent reviewers will conduct study screening, data extraction and risk of bias assessment. Discrepancies will be resolved by consensus or a third reviewer if required. A random effects model will be used to synthesise the results. Alternative synthesis methods will be investigated in instances where a meta-analysis is not appropriate, such as summarising effect estimates, combining *P* values, vote counting based on direction of effect, or synthesis in narrative form.

**Discussion:**

The review will synthesise the evidence on the effect of interventions that address multiple SNAP behaviours in antenatal care and will help researchers, policy-makers and health services to develop and deliver best practice integrated models of antenatal care that have the potential to impact on both the short- and long-term health outcomes for women and their children.

**Systematic review registration:**

PROSPERO CRD42018095315

## Introduction

A woman’s health behaviours during pregnancy have important implications for pregnancy and birth, as well as health outcomes for herself and her child both in the short term and across their life courses [1–6]. Tobacco smoking, poor nutrition, alcohol consumption and low physical activity, collectively referred to as the SNAP risk factors, are common maternal health behaviours associated with adverse health outcomes [7]. Pregnant women with multiple SNAP health behaviours are at even greater risk than those with single health behaviours, with evidence of a possible interactive effect of some health behaviours. For example, alcohol can impair the absorption and availability of maternal nutrient and energy intakes required for foetal development [8]. The co-occurrence of both smoking and alcohol consumption during pregnancy also increases the risk of preterm labour, low birth weight and growth restriction, beyond the individual effects of either smoking or alcohol [9]. Women with SNAP health behaviours in pregnancy are also more likely to continue to engage in these behaviours after pregnancy [10–13], increasing their risk of obesity [14] and chronic diseases including cardiovascular disease, type II diabetes, hypertension, dyslipidaemia, stroke and some cancers [15]. To address these risks, international [16, 17] and national guidelines [18, 19] recommend that pregnant women quit tobacco smoking, consume a diet that is adherent to food group recommendations, abstain from consuming alcohol and engage in 150 min of moderate physical activity each week.

Despite the risks associated with SNAP health behaviours and the guidelines advising pregnant women of recommended health behaviours, the prevalence of SNAP health behaviours in pregnancy is high. Internationally, studies have found that 10–30% of women smoke tobacco during pregnancy [20–24], 97–100% do not meet all pregnancy food group recommendations [25, 26], 20–80% consume alcohol [27, 28] and 53–75% do not meet the recommended 150 min of moderate physical activity each week [29–31]. The clustering of SNAP health behaviours is well established in the general population [32, 33] and among pregnant women specifically [34–38]. In the Canadian Community Health Survey, pregnant women who smoked daily during pregnancy were 2.5 times more likely to have consumed alcohol in pregnancy compared with pregnant women who had never smoked in their life [35]. In the UK Avon Longitudinal Study of Parents and Children (ALSPAC) [39], consuming at least one unit of alcohol each day in the first trimester and consuming alcohol at high risk levels on single occasions (at least four units of alcohol in 1 day) in the first half of pregnancy were both associated with consuming a processed food dietary pattern, characterised by high intakes of processed meats, white bread and fried foods and low intakes of fruit and vegetables. The high prevalence and co-occurrence of adverse SNAP health behaviours in pregnant women supports the need for models of antenatal care that integrate best practice care for multiple health behaviours.

International [16] and national [17–19] antenatal care guidelines recommend that clinicians assess and support pregnant women to improve their SNAP health behaviours throughout the duration of their pregnancy. Health care services that provide antenatal care to women are opportune settings to engage and support pregnant women to improve their health behaviours during the antenatal period. Depending on their model of antenatal care, pregnant women may have an opportunity for regular contact with a range of primary health care clinicians including obstetricians, general practitioners, midwives and Aboriginal health workers and can be referred to other allied health and specialist services for additional behavioural support including dietitians, exercise physiologists, psychologists, smoking cessation counsellors and drug and alcohol specialists. Examining the effectiveness of interventions provided by, or instigated in, antenatal care could inform the delivery of routine integrated models of care for SNAP health behaviours.

Interventions that target co-occurring behavioural risks in pregnancy have potential for greater impact on a range of outcomes for women and their children than interventions addressing single behaviours [40], however research to date has focussed on the latter [40]. Previous reviews targeting co-occurring health behaviours in pregnancy have primarily focused on nutrition and physical activity as related to a specific health outcome, such as gestational weight gain [41]. There is limited evidence to guide health care providers on how to address multiple SNAP health behaviours [34] in integrated care pathways. To our knowledge, no systematic review has provided a synthesis of the field to examine the effect of interventions that address multiple SNAP behaviours on these health behaviours, or related health outcomes. The objective of this review is to determine the effectiveness of interventions delivered as part of antenatal care that aim to improve multiple SNAP behaviours among pregnant women. The secondary aim of this review is to determine if addressing multiple health behaviours results in greater behaviour change than targeting a single health behaviour.

## Methods

The systematic review has been registered with the International Prospective Register of Systematic Reviews (PROSPERO; registration number CRD42018095315). The review protocol was reported in accordance with the Preferred Reporting Items for Systematic Reviews and Meta-Analyses Protocol (PRISMA-P) recommendations [42] (Additional file 1). The methods are consistent with those recommended by the Cochrane Handbook for Systematic Reviews of Interventions Version 6.0 [43].

### Outcomes

The primary outcomes of the review are pregnant women’s SNAP behaviour outcomes: tobacco smoking, nutrition intake, alcohol consumption and physical activity. The secondary outcomes are maternal and/or child health outcomes, and any unintended consequences or adverse outcomes of the intervention.

### Search strategy

Electronic bibliographic databases will be searched for eligible peer reviewed literature, including: MEDLINE (Ovid), EMBASE (Ovid), PsycINFO (Ovid), Maternity and Infant Care (Ovid), Scopus, CINAHL (EBSCOhost) and Cochrane Library (Wiley). The reference lists of all included studies will be screened for other potentially eligible studies. The search strategy will be developed in consultation with a research librarian. There will be no restrictions on the length of the study follow-up period, country of origin or year of study. Only studies published in English will be eligible for consideration.

The strategy will include search terms for participant, intervention, comparator, outcome and study design. The database search term strategy for MEDLINE is described in Additional file 2. The search strategy will be adapted for other databases using appropriate syntax and terminology developed in consultation with a research librarian. The sensitivity of the study design search filter in detecting non-randomised study designs is unknown and it is possible that some such trials may have been missed.

### Eligibility criteria

#### Participants

Studies of pregnant women receiving antenatal care at any time during pregnancy will be included. Studies of women in preconception or postnatal periods are not eligible for inclusion. Studies with interventions that span pregnancy and preconception or postnatal periods will be included only when intervention details and behavioural outcome data is outlined separately for the pregnancy period.

#### Interventions

Studies that include any intervention delivered during antenatal care, or instigated in antenatal care (e.g. referral to another health service for support), that aims to address multiple health behaviours (i.e. more than one SNAP behaviour) during pregnancy will be included. This includes all care provided or instigated in the antenatal care setting regardless of which health professional provided the care (e.g. antenatal clinician or health professional employed as a research personnel to provide care within the study). Antenatal settings can include primary care, hospital antenatal clinics, community antenatal clinics, home antenatal clinics, private obstetrics, midwifery care or allied health services. Studies with interventions that are in settings that are not usual health care providers to women during pregnancy, such as mass media health education campaigns, will be excluded. SNAP health behaviours include tobacco smoking, nutrition, alcohol consumption and physical activity. The intervention could include, but are not limited to, assessing pregnant women’s health behaviour/s through validated and objective instruments, or self-reported behaviours; advising women on the health behaviour recommendations and providing education on the risks associated with adverse SNAP health behaviours during pregnancy; and providing behavioural support directly or offering a referral to other allied health and specialist services for additional support. Interventions only addressing sedentary behaviour will not be included in this review.

#### Comparator

Studies will be included that compare a multiple health behaviour intervention with no-intervention control, wait-list control, usual care, or another active intervention addressing one health behaviour (e.g. alcohol only).

#### Outcomes

Studies that include any measure of pregnant women's modifiable health behaviours, including tobacco smoking and/or cessation, nutrition intakes (e.g. reported as dietary intakes for energy, macronutrients or micronutrients; adherence to food or nutrient guidelines; or overall diet quality scores), alcohol consumption and/or abstinence and physical activity will be included. Outcome data can be collected via any data collection method (e.g. self-report, observation, or objective measures) including audits of service or medical records such as patient pregnancy records.

#### Types of studies

Systematic review of randomised controlled trials (RCTs), RCTs, cluster RCTs, stepped-wedge RCTs (or staggered enrolment trials) and non-randomised controlled trials will be eligible for inclusion. Studies with designs without a parallel control group or another active intervention addressing one SNAP health behaviour (e.g. pre-post studies or historic control groups) will be excluded.

### Data collection and analysis

#### Study selection

The titles and abstracts identified through the search strategy will be screened for eligibility by two independent reviewers using the eligibility criteria above. Studies that do not meet the criteria will be excluded. The full texts of all remaining studies will be reviewed by two independent reviewers, and the reason for exclusion will be recorded. Any discrepancies regarding study eligibility will be resolved through discussion and consensus between the two reviewers, or if required, consultation with a third reviewer. A reviewer will contact the study authors for clarification where there is insufficient information to determine study eligibility. If sufficient information is still unavailable, the study will be excluded from the review. Screening will be managed in Covidence (www.covidence.org).

#### Data extraction

Data from the eligible studies will be extracted by two independent reviewers. Data will be extracted using a standardised electronic form consistent with data collection items recommended by the Cochrane Handbook for Systematic Reviews of Interventions [43]. The form will be piloted prior to use. Any discrepancies in data extracted will be resolved by consensus between the two reviewers, or consultation with a third reviewer if required. Reviewers extracting data will not be blind to author or journal information.

The following information will be extracted:

- Study characteristics: authors (including contact details), article citation, date of publication, country of study, aim of study, dates intervention undertaken, participant characteristics (i.e. number of participants, age, body mass index, ethnicity, sociodemographic information, parity, clinical conditions, weeks’ gestation), sample size, missing participants, study design, number of experimental groups, antenatal service setting (e.g. primary care, hospital antenatal clinics, home visit antenatal services or allied health) and information to assess risk of bias
- Intervention characteristics: SNAP behaviour change targeted, duration of intervention, number of contacts, theoretical underpinning of intervention and comparator, intervention content, mode of delivery (e.g. individual or group, in-person, online, telephone) and profession of intervention deliverer (e.g. midwife, obstetrician, dietitian, general practitioner, researcher)
- Outcomes: SNAP behaviour outcomes (smoking, nutrition, alcohol, physical activity) including definitions, maternal and/or infant health outcomes (i.e. health outcomes measured such as gestational weight gain, gestational diabetes, gestational hypertension, pre-eclampsia, preterm birth, low birth weight, macrosomia, foetal alcohol spectrum disorder and child and maternal obesity) including definitions, duration of follow-up, data collection method, name of tool, validity of measures used, scale of measure, number of participants per comparison group at each time point and effect size and measures of outcome variability
- Other: Key conclusions of the study authors, any unintended consequences or adverse outcomes, sources of funding and any potential conflicts of interest

#### Assessment of risk of bias

The risk of bias of the included studies will be assessed by two independent reviewers for both primary and secondary outcomes. The Cochrane Risk of Bias Tool, RoB2 [43], will be used to assess study characteristics for each RCT that is included in the review, including (i) bias from the randomisation process, (ii) bias from deviations from intended interventions, (iii) bias from missing outcome data, (iv) bias in the measurement of the outcome, and (v) bias in the selection of the reported results. An additional criteria will be used to assess risk of bias in cluster RCTs: (vi) bias arising from identification or recruitment of individual participants within clusters [43]. For included studies of non-randomised trial design, risk of bias will be assessed using the Risk Of Bias In Non-randomized Studies of Interventions (ROBINS-I) tool [44]. A consensus approach between the two reviewers will be used to resolve any assessment discrepancies, or a third reviewer with expertise in review methodology will be consulted.

#### GRADE

The GRADE approach [45] will be used to assess the overall quality of the evidence for the each of the four behavioural primary outcomes. Two independent reviewers will conduct the assessment, with discrepancies resolved through discussion and consensus between the two reviewers, or consultation with a third reviewer. Quality ratings ranging from ‘very low’ to ‘high’ will be assigned to each study.

#### Measures of treatment effect

Where possible, trial data will be combined and reported using meta-analyses using the standard estimation of (1) risk ratio (RR) and 95% confidence intervals (CI) for dichotomous outcome variables, and (2) mean differences (MDs) or standardised mean differences (SMDs) and 95% CIs for continuous outcome variables. We will use SMDs when studies report the same outcome and a comparable, but not identical, measure. In instances where a meta-analysis is not appropriate, alternative synthesis methods will be investigated as recommended by the Cochrane Handbook for Systematic Reviews of Interventions [43], such as summarising effect estimates, combining *P* values, vote counting based on direction of effect, or synthesis in narrative form.

#### Unit of analysis issues

We expect to identify individually randomised and cluster-randomised studies for this review. We will examine cluster trials for unit of analysis errors. Where unit of analysis errors are identified, we will attempt to correct them prior to including the data in pooled analyses. For cluster design trials, individual level behaviour data adjusting for clusters using an intracluster correlation co-efficient (ICC) will be extracted. Study authors will be contacted to provide the ICCs for trials where the effects of clustering have not been reported. Where study authors are unable to provide ICCs, a mean ICC will be estimated from reported ICCs of included studies with similar behavioural outcome data and used to calculate effective sample sizes.

#### Dealing with missing data

Study outcome data analysed using the intention to treat (ITT) principle will be preferentially extracted over other outcome data (e.g. analysed using a completer’s analysis), unless no ITT data is available. We will contact study authors to obtain missing data where it occurs. We will conduct sensitivity analysis excluding trials with high levels of missing outcome data (> 30%).

#### Assessment of heterogeneity

Clinical, methodological and statistical heterogeneity among studies will be considered when deciding if it is appropriate to perform a meta-analysis. Clinical heterogeneity will be firstly assessed to determine if the studies vary in intervention and outcomes reported. Variance in study design, outcome measure tools and risk of bias among the studies will be considered for methodological heterogeneity. Statistical heterogeneity of included studies will be examined using visual inspection of forest plots and statistically quantified by calculating the *I*^2^ statistic [43]. An *I*^2^ statistic of > 50% is considered to be substantial heterogeneity. We will perform subgroup analyses to identify the source of heterogeneity in such circumstances. Consensus will be sought between review authors regarding the appropriateness of a meta-analysis [43].

#### Data synthesis

Data from randomised and non-randomised study designs will be synthesised separately. Outcomes will be reported by intervention characteristics. Assuming the presence of heterogeneity, the primary and secondary outcomes will be combined in a meta-analysis using a random effects model (and the DerSimonian and Laird method to estimate the between-study variance) through the Review Manager 5 (RevMan 5) soLware (Review Manager 2014) and reported as a RR, MD, and SMD. Alternative synthesis methods will be conducted as recommended by the Cochrane Handbook for Systematic Reviews of Interventions [43] if studies cannot be combined in a meta-analysis.

Where studies report multiple of the same behavioural outcomes (e.g. for smoking cessation) using different data collection methods (e.g. self-report measures by women, health professional self-report, researcher observations or medical record audits), the outcome that is deemed to represent the most valid measure and/or for which the longest follow-up or most complete data is reported will be used. Only intervention and control groups that meet the eligibility criteria from multiple arm studies will be included in data synthesis. If a study contains multiple intervention or control arms that are all eligible for inclusion, a decision will be made to either (i) collapse all intervention and/or control arms into single pair wise comparisons or (ii) conduct bivariate analyses with all eligible arms included and adjust for the repeated inclusion of the same intervention and/or control arm.

#### Assessment of reporting biases

The methods and analyses of published studies will be compared to trial protocols and registers to identify instances of potential selective reporting. Funnel plots will be generated for each outcome to determine potential publication bias for meta-analyses including 10 or more studies.

#### Sensitivity analysis

Sensitivity analyses will be conducted for each of the SNAP behaviour outcomes where there are sufficient studies. This will be performed by removing studies with an overall high risk of bias to examine their impact on the effect estimate.

## Discussion

This systematic review will determine the effectiveness of interventions in antenatal care that aim to improve multiple SNAP health behaviours among pregnant women. The review will be of value to researchers, policy-makers and health services providing antenatal care. The findings will inform the development and delivery of effective integrated models of care for multiple health behaviours that have the potential to impact on short- and long-term health outcomes for women and children.

## Supplementary information

**Additional file 1.** PRISMA-P 2015 Checklist.

**Additional file 2.** Search Term Strategy.

## Data Availability

Not applicable.

## Abbreviations

ALSPAC: Avon Longitudinal Study of Parents and Children
CI: Confidence interval
ICC: Intracluster correlation co-efficient
ITT: Intention to treat
MDs: Mean differences
PRISMA-P: Preferred Reporting Items for Systematic Reviews and Meta-Analyses Protocol
PROSPERO: International Prospective Register of Systematic Reviews
RCTs: Randomised controlled trials
RR: Risk ratio
SMDs: Standardised mean differences
SNAP: Smoking, Nutrition, Alcohol, Physical activity
