# Supplementary material for: Are antenatal interventions effective in improving multiple health behaviours among pregnant women? A systematic review protocol
Source: Syst Rev. 2020 Sep 2;9:204. doi: 10.1186/s13643-020-01453-z (PMC7469269; doi:10.1186/s13643-020-01453-z)
Supplement: Supplementary file 2 — Additional file 2. Search Term Strategy. [file 13643_2020_1453_MOESM2_ESM.docx]

**Appendix 2. Search Term Strategy**

1. Pregnan*.tw.

2. Matern*.tw. or Maternal Behaviour/

3. Gestation*.tw.

4. 1 or 2 or 3

5. Prenatal Care/ or (antenatal or prenatal).tw.

6. (Healthcare or healthcare).tw. or “Delivery of Health care”/

7. Maternal Health Services/ or Maternity Health Services.tw. or Family Planning Services/

8. Midwifery/ or Midwi*.tw. or Nurse Midwives/

9. Physicians, Family/ or Family Practice/ or General Practitioner*.tw. or General Practice/ or General Practitioners/

10. Obstetric*.tw. or Obstetrics/

11. ((Health or healthcare) adj2 (professional* or worker*)).mp.

12. 5 or 6 or 7 or 8 or 9 or 10 or 11

13. Controlled Clinical Trial.tw. or Controlled Clinical Trial/

14. Randomi?ed.tw

15. Clinical Trials as Topic/ or Placebo.tw.

16. Randomly.tw.

17. Randomised Controlled Trial/ or Clinical Trial/ or Trial.tw

18. Groups.tw.

19. Step*wedge.tw.

20. Meta-Analysis as Topic/ or Systematic Review.tw.

21. 13 or 14 or 15 or 16 or 17 or 18 or 19 or 20

22. Behavio* adj2 (health or risk* or multiple or multi or multi-risk)

23. Gestational weight gain.tw.

24. Pregnancy Weight Gain.tw.

25. Weight gain.tw. or Weight Gain/

26. Fetal Alcohol Spectrum Disorders/ or Fetal Alcohol Spectrum Disorder*.tw. or FASD.tw.

27. Alcohol Abstinence/ or Alcohol*.tw.

28. Drinking behaviour/ or exp Alcohol Drinking/ or Drinking/ or drinking.tw. or Binge Drinking/

29. Ethanol.tw. or Ethanol/

30. (Smok* adj2 (prevent* or reduc* or cessation or cease* or cigarette or tobacco)).mp.

31. Tobacco use/ or Tobacco/ or Tobacco Smoking/ or Tobacco use cessation/ or Tobacco.tw.

32. Cigarette*.tw. or Tobacco Products/

33. Smoking Cessation/ or Nicotine Replacement Therapy.tw. or Smoking/

34. NRT.tw.

35. Nutrition.tw.

36. Diet.tw. or Diet/ or Heathy Diet/

37. Food.tw. or Food/ or “Diet, food and nutrition”/

38. Eat*.tw. or Eating/

39. Energy Intake.tw. or Energy Intake/

40. Physical Activit*.tw.

41. Exercise/ or Exercise.tw.

42. Sedentary Lifestyle/ or Physical Inactivit*.tw.

43. (Sedentary adj2 (Behavio* or Lifestyle)).mp.

44. Aerobic.tw.

46. Fitness.tw. or Physical Fitness/

47. Physical exercise.tw.

48. Motor activity.tw. or Motor Activity/

49. Sitting Time.tw.

50. 22 or 23 or 24 or 25

51. 26 or 27 or 28 or 29

52. 30 or 31 or 32 or 33 or 34

53. 35 or 36 or 37 or 38 or 39

54. 40 or 41 or 42 or 43 or 44 or 45 or 46 or 47 of 48 or 49

55. 51 and (52 or 53 or 54)

56. 52 and (51 or 53 or 54)

57. 53 and (51 or 52 or 54)

58. 54 and (51 or 52 or 53)

59. 50 or 55 or 56 or 57 or 58

60. 4 and 12 and 21 and 59
